# Limits for the therapeutic application of the analytical anisotropic algorithm in the context of ablative lung radiotherapy near the minima of lung density and tumor size

**DOI:** 10.1002/acm2.13634

**Published:** 2022-05-09

**Authors:** Eric Lobb, Ahpa Plypoo

**Affiliations:** ^1^ Department of Radiation Oncology Ascension NE Wisconsin – St. Elizabeth Hospital Appleton Wisconsin USA

**Keywords:** heterogeneity corrections, SABR, SBRT, stereotactic ablative radiation therapy, treatment planning algorithm

## Abstract

**Purpose:**

To systematically investigate the performance of the analytical anisotropic algorithm (AAA) within the extremes of small tumor volumes and near‐minimum lung and tumor tissue densities in order to identify combinations of these parameters where the use of AAA could result in a therapeutically unacceptable loss of tumor coverage on an energy and fractionation‐specific basis.

**Methods:**

Clinically appropriate volumetric modulated arc therapy (VMAT) treatment plans were generated with AAA for 180 unique combinations of lung density (0.05–0.30 g/cm^3^), tumor density (0.30–1.00 g/cm^3^), tumor diameter (0.5–2.5 cm), and beam energy (6 and 10 MV) and recomputed using the AcurosXB algorithm. Regression analysis was used to identify the strongest predictors of a reduction in biologically effective dose at a clinically relevant level (100 Gy BED10) for commonly utilized 1–5 fraction treatment regimens. Measurements were performed within a phantom mimicking the lower extremes of lung and tumor densities to validate AcurosXB as the approximate ground truth within these scenarios.

**Results:**

The strongest predictors of a statistically significant reduction in tumor coverage were lung density ≤0.15 g/cm^3^, tumor diameter ≤10 mm, tumor density equal to 0.30 g/cm^3^, and a beam energy of 10 MV. Overestimation of clinical target volume (CTV) D95% and CTV V100Gy (BED10) by AAA can exceed 30%–40% in some scenarios. Measurements supported AcurosXB as highly accurate even for these challenging scenarios.

**Conclusions:**

The accuracy of AAA rapidly diminishes near the minima of clinical lung density, particularly in combination with small tumors and when using a photon energy of 10 MV. The magnitude of the effect can be more dramatic than previously reported data suggests and could potentially compromise the ablative qualities of treatments performed within these environments, particularly with less aggressive fractionation approaches.

## 1 INTRODUCTION

Stereotactic body radiation therapy (SBRT) or stereotactic ablative radiotherapy (SABR) is a commonly utilized technique for the therapeutic management of early‐stage non‐small cell lung cancer. It combines precise tumor targeting with the delivery of an ablative dose of highly conformal radiation that rapidly falls off at the target boundary to minimize treatment‐related toxicity, generally delivered in five or fewer total treatment sessions.^1^

Among several technical factors that must be considered in the safe utilization of the SABR technique are those related to the choice of dose calculation algorithm utilized in the treatment planning process. Users of the Eclipse treatment planning system (Varian Medical Systems, Palo Alto, CA) have two primary options for modern photon dose calculation algorithms: the analytical anisotropic algorithm (AAA) and the AcurosXB algorithm. Briefly, AAA is a 3D pencil beam convolution–superposition algorithm with anisotropic tissue heterogeneity handling via multidirectional photon scatter kernels. AcurosXB is a numerical solver of the Linear Boltzmann Transport Equation that describes the interaction of radiation particles as they move through matter, handling tissue heterogeneities via explicit modeling of the physical radiation interactions. Detailed specifications of each algorithm are available within the Varian algorithm reference guides.^2^

Ablative radiotherapy to lung tumors combines several challenges in accurate dose calculation, chief among them being the use of typically small treatment fields within a highly heterogenous tissue environment. Planning target volumes (PTVs) typically include a margin of lung tissue surrounding a soft‐tissue tumor with a dramatic density transition, and the loss of charged particle equilibrium within the tumor volume itself can result in severe underdosage if not accounted for appropriately.^3^ For relatively larger tumors, this may be of concern primarily near the lung and tumor interface, but for small tumors, this effect can encompass most or all of the targeted volume.

Both AAA and AcurosXB are considered to have acceptable heterogeneity handling capabilities for institutional credentialing of ablative lung radiotherapy clinical trials.^4^ Data on the current extent of AAA utilization within clinics performing lung SABR is not readily available, but it has been shown by Glenn et al. that the self‐reported use of AAA was approximately 3.5 times greater than AcurosXB as recently as 2018–2019, suggesting that AAA is still commonly utilized within the radiation oncology community, likely including many centers that do perform lung SABR.^5^

AAA and AcurosXB have been extensively reported on in the literature through intercomparisons of algorithm output under various calculation conditions, comparison with robust Monte Carlo techniques, and through direct comparisons with measurements. Within the context of ablative lung radiotherapy, the generalized findings are that AAA tends to overestimate the delivered dose to the target volume by approximately 10% or less, with the severity of overestimation increasing as tumor size decreases.^6^, ^7^, ^8^, ^9^, ^10^, ^11^, ^12^, ^13^, ^14^, ^15^, ^16^

However, our own institutional experience is that there is a subset of lung SABR patients for whom this assumption of only a modest overestimation of dose by AAA is not valid. Patients who present with atypically low lung densities (due to severe pulmonary emphysema or cystic disease local to the lung tumor, for example) routinely demonstrate a much larger than expected difference in calculated dose between the two algorithms, well above the commonly reported 10% value. We echo the findings of other researchers that the magnitude of the effect is exacerbated for small tumors and hypothesize that low tumor density may also be a contributing factor.

Our internal experience is similarly reported in Liu et al.’s study of 77 lung SABR patients where they found correlation between AAA dose calculation accuracy and forced expiratory volume in 1 second (FEV1),^16^ which itself can be correlated with lung density.^17^ Specifically, the authors reported that lower FEV1 values (indicative of reduced pulmonary function) correspond with larger discrepancies in tumor dose between AAA and AcurosXB, with one presented case showing a nearly 13% reduction in PTV coverage at the prescribed dose level.

Currently, there does not appear to be a systematic investigation of the performance of the AAA specific to the clinically possible extremes of lung density, tumor volume, and tumor density. Our goal is to fill this gap and identify clinical scenarios that could compromise the therapeutic intent of a prescribed course of ablative lung radiotherapy when planned using AAA. Further, we intend to determine this information as a function of common fractionation schemes and common photon beam energies utilized for lung SABR treatment delivery.

## 2 MATERIALS AND METHODS

### 2.1 AcurosXB validation at density minima

**FIGURE 1:**
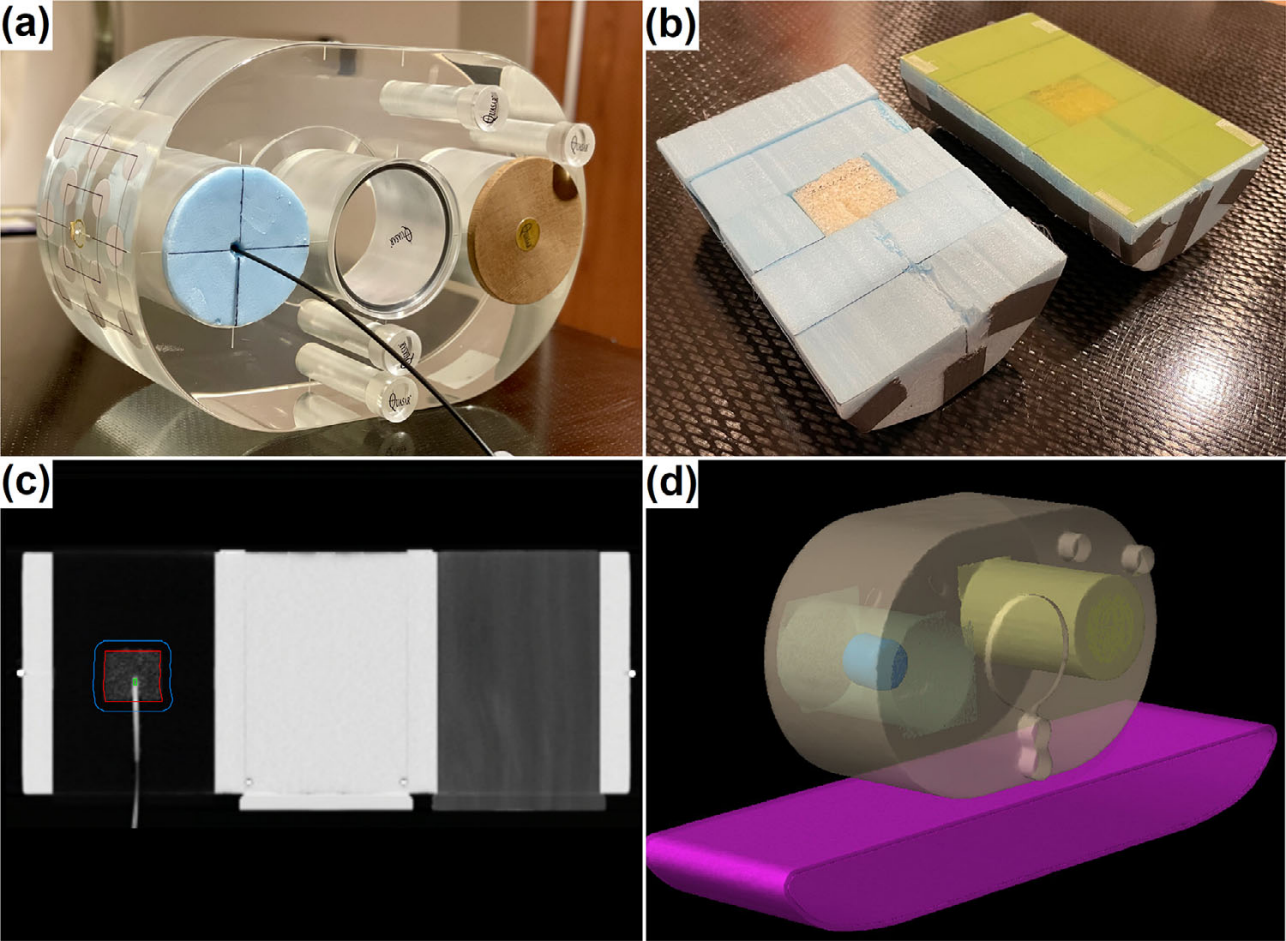
Phantom geometry used for film and point‐dose measurements: (a) assembled phantom with inserted scintillator, (b) bisected “lung” and “tumor” inserts with film, (c) coronal view of CT dataset with scintillator inserted into the tumor surrogate, (d) TPS model of phantom and treatment couch

In this study, a number of treatment plans generated using AAA are recomputed using AcurosXB in order to expose an underlying dose distribution that is closer to the physical reality. Congruency of AcurosXB with measurement and Monte Carlo simulation has been extensively documented for heterogeneous tissue environments, including applications in lung radiotherapy. However, the validation of clinical treatment plans using AcurosXB specifically for scenarios involving densities at extreme minima is limited. As the validity of much of the data in this study relies on the AcurosXB calculations representing an approximate “ground truth” under these scenarios, validation measurements at those extremes were warranted. Descriptions of the measurement techniques follow in Sections 2.1.1 and 2.1.2, with Figure 1 showing the geometry of the measurement phantom.

All treatment planning calculations in this study were performed with either the AAA 15.5.11 or AcurosXB 15.5.11 algorithms using a 1‐mm dose calculation grid and with heterogeneity corrections enabled. For calculations, using AcurosXB dose was reported to the medium. The slice thickness of all computed tomography (CT) datasets used in this study was 1 mm. All fluence optimizations relevant to this study were performed using the Photon Optimizer 15.5.11 with structure resolution set to Fine, Convergence Optimization Mode set to On, Dose Calculation Resolution of the optimization engine set to High, and Intermediate Dose performed.

#### 2.1.1 Point‐dose verification

The phantom used for point‐dose verification was the QUASAR Respiratory Motion Phantom (Modus Medical Devices, London, Ontario), which consists of an acrylic body (30‐cm width, 20‐cm height, and 12‐cm length) that accepts cylindrical inserts centrally and laterally. For this study, the left lateral opening was filled with a moderately low‐density cedar insert, and the central opening was filled with an acrylic plug.

The right lateral opening was filled with a custom foam insert of density 0.04 g/cm^3^, designed to mimic a near minimum‐possible density of lung tissue. Embedded centrally within the foam was a 2.5‐cm long and 2.5‐cm diameter tumor surrogate constructed from 0.31‐g/cm^3^ LN‐300 tissue characterization material (Sun Nuclear Corporation, Melbourne FL), approximately matching the minimum lung tumor density encountered in our institution. The tumor surrogate was drilled to accept a 1 mm × 1 mm Exradin W2 plastic scintillating detector (Standard Imaging Inc., Middleton WI) for the point verification of dose at the tumor center.

The LN‐300 tumor surrogate was labeled clinical target volume (CTV) and expanded uniformly by 5 mm to create the PTV. Optimization parameters were selected to deliver 1250 cGy to 95% of the PTV using a VMAT technique with a pair of 225‐degree coplanar partial arcs, spanning from the phantom posterior to 45 degrees contralaterally, with isocenter positioned in the centroid of the CTV. The optimized treatment plan had initial dose calculation performed using AAA 15.5.11 with a 1‐mm isotropic calculation grid and was then recalculated using identical parameters with AcurosXB 15.5.11.

Calibration of the scintillator was performed immediately prior to treatment delivery using the manufacturer's suggested workflow for Cerenkov light ratio determination along with an absolute dose cross‐calibration against an ADCL‐calibrated Farmer‐type ionization chamber. The measured dose from plan delivery was directly compared against the mean dose to the scintillating volume calculated by the treatment planning system.

#### 2.1.2 2D dose verification

Radiochromic EBT‐XD film (Ashland Advanced Materials, Bridgewater, NJ) was utilized for spatial dose verification in the coronal plane of the phantom. The phantom geometry was similar to the described geometry for point dose measurement, with the difference being that the tumor surrogate was bisected for the placement of an 8 × 12‐cm^2^ sheet of the film. Therefore, the measurement plane consisted of the coronal cross‐section of the LN‐300 tumor surrogate and low‐density foam lung surrogate. Calibration and handling of the film was performed consistent with published recommendations.^18^

The irradiated film was digitized using an Epson 10000XL flatbed scanner (Epson America Inc., Long Beach, CA) at 300‐dpi and 48‐bit color depth. Optical density was extracted from the RGB file using the green color channel and imported into RIT V6.9 (Radiological Imaging Technology Inc., Colorado Springs, CO) for the application of the batch‐specific film calibration and registration with the exported dose planes.

### 2.2 Simulated tissue characteristics

To systematically evaluate the effect of tissue characteristics on the difference in calculated dose between AAA and AcurosXB, a representative clinical lung SABR case was selected to serve as the CT dataset for simulated tests. A synthetic CTV was centrally placed within the right mid‐lung and was represented as a homogeneous spherical contour with diameters of 0.5, 1.0, 1.5, 2.0, and 2.5 cm. The ipsilateral lung was assigned densities of 0.05, 0.10, 0.15, 0.20, 0.25, and 0.30 g/cm^3^ with a fixed material assignment of “Lung Tissue.” The CTV was separately assigned densities of 0.30, 0.65, and 1.00 g/cm^3^. The CTV was uniformly expanded by 5 mm to create the PTV.

For each combination of lung density, tumor density, and tumor diameter, a clinically appropriate VMAT treatment plan was optimized and calculated using AAA with a pair of 225‐degree coplanar arcs and isocenter positioned in the CTV centroid, normalized such that the dose delivered to 95% of the PTV (D95%) was equal to 100% of the prescribed dose (1250 cGy) and then recalculated with fixed parameters using AcurosXB. This process was performed for both 6 and 10‐MV photon energies, resulting in 180 unique scenarios for each algorithm.

Dose coverage statistics for the CTV and PTV were recorded for each of the 180 treatment plans and were tabulated as both percentages and biologically effective doses (BED) with an assumed alpha‐beta ratio of 10 (BED10). It should be noted that reduction in tumor coverage by an arbitrary percentage cannot necessarily be assumed to compromise the efficacy of the treatment. For example, if a plan generated using AAA yields a CTV that is covered by the 120% isodose line but the true dose distribution reveals that the CTV is actually covered by the 95% isodose line, this may still be a sufficiently ablative dose to achieve the desired therapeutic outcome. The point at which the dose reduction becomes unacceptable depends on the nominally prescribed BED (BED_RX_), which is a function of the fractionation scheme being utilized.

In order to address this, the percentage of CTV receiving a commonly utilized minimum BED for ablative treatment of lung tumors (100‐Gy BED10)^19^, ^20^, ^21^, ^22^, ^23^, ^24^, ^25^ was tabulated for a number of common fractionations: 3400 cGy/1 Fx (BED_RX_ = 149.6 Gy), 5400 cGy/3 Fx (BED_RX_ = 151.2 Gy), 5000 cGy/4 Fx (BED_RX_ = 112.5 Gy), 4800 cGy/4 Fx (BED_RX_ = 105.6 Gy), 6000 cGy/5 Fx (BED_RX_ = 132.0 Gy), and 5000 cGy/5 Fx (BED_RX_ = 100.0 Gy). This data was extracted from the same set of 180 unique combinations of lung density, tumor density, and tumor size previously described, with consistent methodology for treatment plan design using AAA and recalculation using AcurosXB. The resulting data was expected to highlight the relative sensitivity of these fractionation schemes to overprediction of dose by AAA specifically in the context of achieving 100 Gy (BED10) coverage of the tumor.

### 2.3 Application to clinical cases

In order to validate the applicability of the data derived from the simulated tissue characteristic tests to clinical CT datasets and their associated treatment plans, a sample of five clinical lung SBRT plans with unaltered anatomy were similarly evaluated for changes in coverage metrics between AAA and AcurosXB. Recent cases with mean local lung density <0.20 g/cm^3^ were replanned using AAA and subsequently recalculated using AcurosXB using the same methodology previously described in this study. The resulting changes in CTV and PTV D95% values were tabulated against those predicted by the set of data generated from the simulated tests.

Lung tumor location in these clinical cases was the periphery of the right middle lobe (Cases 1 and 2), the periphery of the left middle lobe (Case 3), centrally within the left middle lobe (Case 4), and the periphery of the right upper lobe (Case 5). All five cases involved patients with diagnosed pulmonary diffuse or bullous emphysema.

### 2.4 Institutional patient lung density distribution

In order to determine the relevance of each lung density data point within our patient population, lung density statistics were collected from our most recent 55 lung SABR patients. For each patient, lung density was determined for the treated lung, excluding gross tumor volume and major central vasculature, using an automated region‐growing segmentation utility that flood‐fills the lung with an upper HU limit of −400 HU, returns the mean HU value, and converts it to physical density using our clinical CT calibration tables.

## 3 RESULTS

### 3.1 AcurosXB validation at density minima

#### 3.1.1 Point‐dose verification

For 6 MV, the measured dose using the scintillating detector was 1362.4 cGy and the calculated dose was 1397.2 and 1357.0 cGy for AAA and AcurosXB, respectively. AAA overpredicted the delivered dose to the tumor center by 2.6%, whereas AcurosXB underpredicted the delivered dose by 0.4%. Similarly for the 10‐MV plan, the measured dose was 1322.2 cGy and the calculated dose was 1448.0 cGy for AAA (9.5% overprediction) and 1310.1 cGy for AcurosXB (0.9% underprediction).

#### 3.1.2 2D dose verification

**FIGURE 2:**
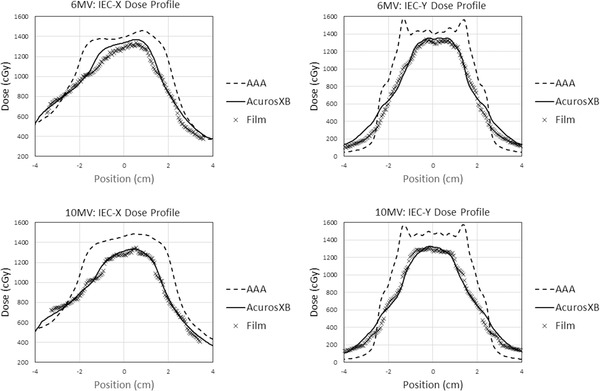
Film measurement results along the IEC‐*X* and IEC‐*Y* axes for 6 MV (top) and 10 MV (bottom)

Measured coronal film profiles in the IEC‐*X* and IEC‐*Y* directions are overlaid with computed AAA and AcurosXB dose profiles in Figure 2. AcurosXB exhibited uniformly superior agreement with measurement in both central dose magnitude and profile shape, with a mean magnitude of percentage dose difference in the 6‐MV plan of 2.83% ± 2.56% for AcurosXB compared to 13.13% ± 7.73% for AAA. For 10 MV, these results were 2.47% ± 2.04% for AcurosXB compared to 14.44% ± 7.96% for AAA.

### 3.2 Simulated tissue characteristics

**FIGURE 3:**
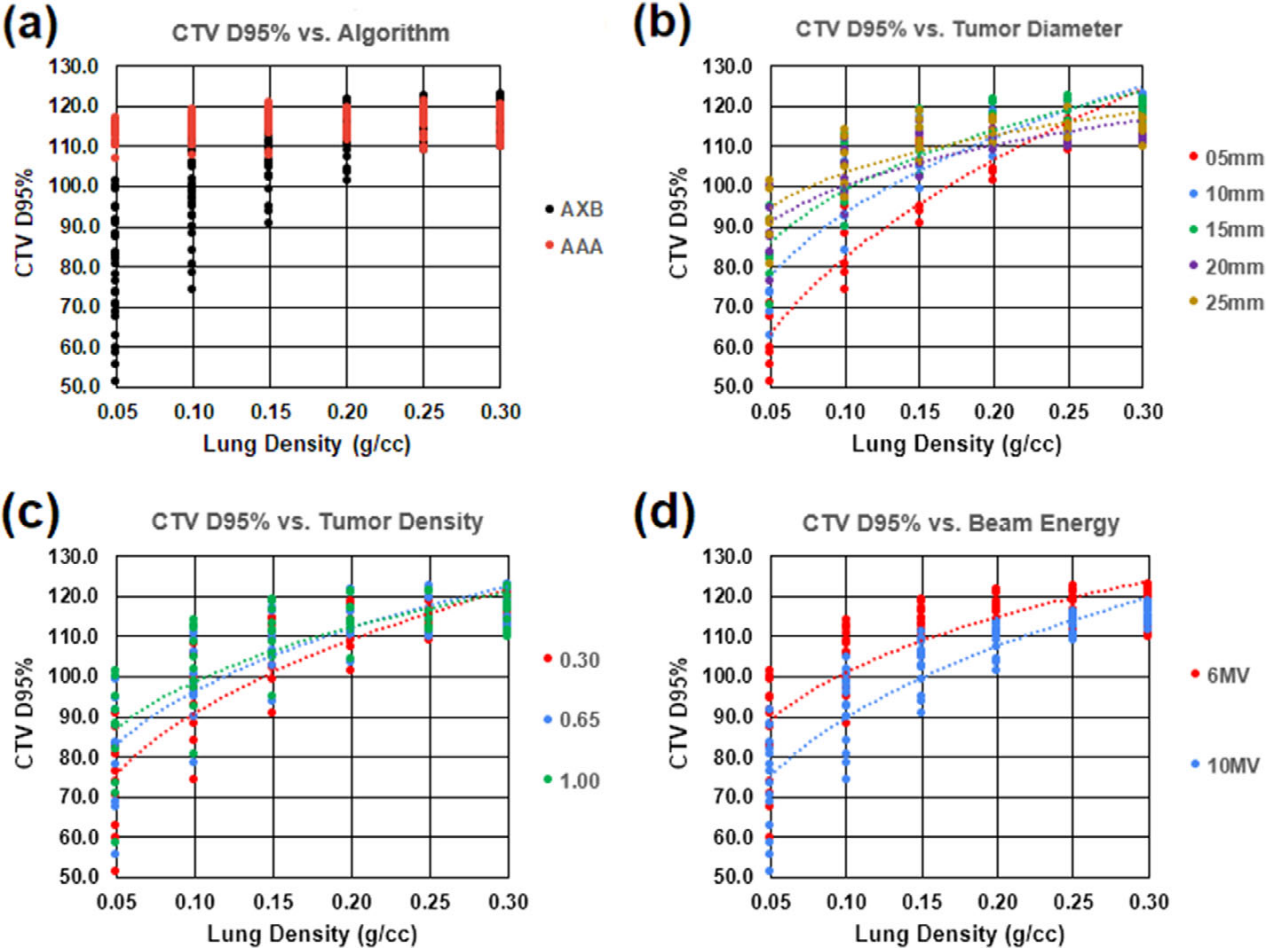
Plots of CTV D95% values for 180 AAA‐generated treatment plans showing (a) data points stratified by algorithm, (b) AcurosXB data points stratified by CTV diameter, (c) AcurosXB data points stratified by tumor density, and (d) AcurosXB data points stratified by beam energy. AAA data points are only included in (a) for comparative purposes and have been removed from (b), (c), and (d). AAA, analytical anisotropic algorithm; CTV, clinical target volume

Plots of CTV D95% values for all tissue characteristic scenarios are provided in Figure 3, with subplots highlighting specific data features and their associated trends. Figure 3a presents the dataset as a function of lung density and stratified by calculation algorithm (AAA base plan data points and AcurosXB recalculated data points). For reference, the entire baseline AAA dataset had a mean CTV D95% result of 116.0% ± 5.8% (2*σ*). Figure 3b–d presents only the AcurosXB data points and stratifies the data based on tumor diameter, tumor density, and beam energy, respectively.

Regression analysis reveals that the strongest predictors of a statistically significant reduction in tumor coverage relative to treatment planning prediction were lung density ≤0.15 g/cm^3^ (*p* < 0.001), tumor diameter ≤10 mm (*p* < 0.001), tumor density equal to 0.30 g/cm^3^ (*p* < 0.001), and a beam energy of 10 MV (*p* < 0.001). Of the 38 data points with CTV D90% ≤95%, 100% were associated with a lung density of ≤0.15 g/cm^3^, 58% with tumor diameter ≤10 mm, 40% with tumor density of 0.30 g/cm^3^, and 66% with a beam energy of 10 MV.

**FIGURE 4:**
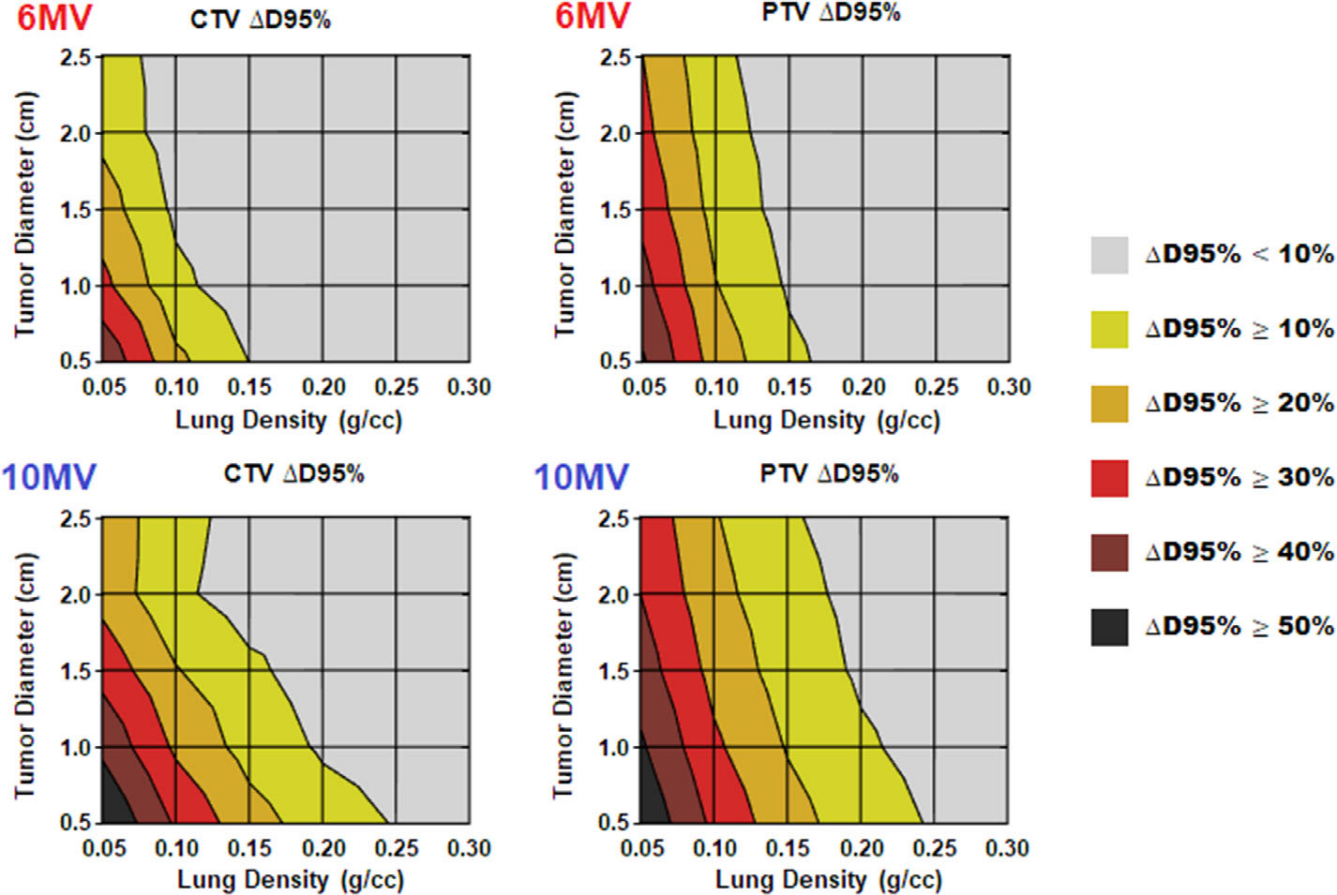
Change in D95% values when recalculating AAA‐designed treatment plans using AcurosXB as a function of lung density and tumor diameter. AAA, analytical anisotropic algorithm

Energy‐specific contour plots showing changes in D95% values for the CTV and PTV between AAA and AcurosXB are shown in Figure 4 as a function of lung density and tumor diameter. For a 6‐MV photon energy, the agreement between the algorithms is within 10% for both the CTV and PTV when lung density is ≥0.15 g/cm^3^. Below 0.15 g/cm^3^, there is a sharp divergence between the algorithms, with discrepancies exceeding 30% when lung density and tumor diameter are both near their minimum values. For 10 MV, the magnitude of dose overestimation by AAA is greater overall with discrepancies ≥10% beginning for the smallest tumors just below a lung density of 0.25 g/cm^3^ and increasing to ≥40% as lung density decreases to 0.10 g/cm^3^.

**FIGURE 5:**
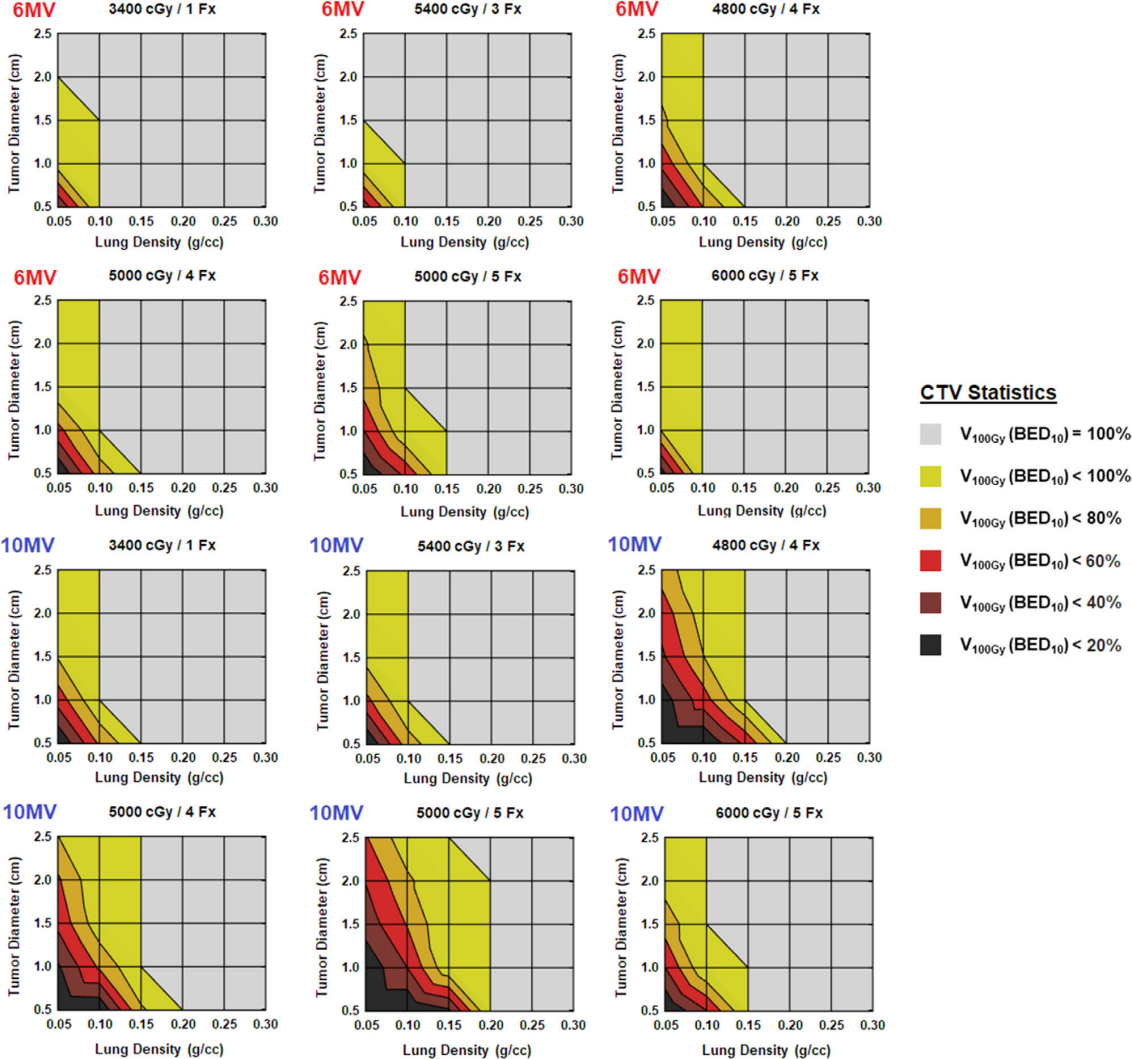
Percentage of CTV receiving 100 Gy (BED10) as a function of lung density and tumor diameter for various fractionations. Values here reflect treatment plans initially designed using AAA (95% PTV coverage at the displayed prescribed dose level) and subsequently recalculated with AcurosXB using identical parameters. AAA, analytical anisotropic algorithm; CTV, clinical target volume; PTV, planning target volume

Energy‐specific contour plots of CTV V100Gy (BED10) values for multiple fractionation schemes are shown in Figure 5. For all fractionations, there is a variable sized region in these plots where the percentage of CTV receiving a BED10 of 100 Gy falls below the full tumor volume. Various reasonably common combinations of lung density, tumor diameter, and fractionation result in 100‐Gy (BED10) tumor coverage values of ≤80%, and there are other less common but still encounterable combinations where this value decreases to 60% or lower.

### 3.3 Application to clinical cases

Changes in CTV and PTV D95% values between AAA and AcurosXB for a sample of recent 10‐MV FFF clinical cases is provided in Table 1. All results for CTV ∆D95% and all but two results for PTV ∆D95% fell within the range of values predicted by the simulated tests. The two outlier results were both slightly above the upper end of the predicted range (51.9 vs. 50.8 and 16.0 vs. 15.5), and most other results trended near the upper end of the predicted range as well, indicating that tumor dose overestimation by AAA in clinical cases may be slightly more pronounced than in the set of simulated tests described in Section 3.2.

**TABLE 1:** Change in CTV and PTV D95% values between AAA and AcurosXB for a sample of recent clinical treatment plans compared to predicted result ranges

Application of results to clinical datasets							
Case	*ρ*‐Lung (g/cm^3^)	*ρ*‐GTV (g/cm^3^)	GTV‐diam (mm)	CTV ∆D95% predicted	CTV ∆D95% actual	PTV ∆D95% predicted	PTV ∆D95% actual
1	0.07	0.98	10	24.8–42.9	40.5	31.4–50.8	51.9
2	0.11	0.81	19	1.8–9.0	3.9	13.1–22.6	17.3
3	0.13	1.00	09	19.4–29.8	26.9	25.4–32.9	29.0
4	0.13	0.92	14	13.1–26.8	26.3	20.7–31.7	28.8
5	0.17	0.76	15	3.5–11	9.2	8.7–15.5	16.0

Abbreviations: AAA, analytical anisotropic algorithm; GTV, gross tumor volume; CTV, clinical target volume; PTV, planning target volume.

### 3.4 Lung density distribution

**FIGURE 6:**
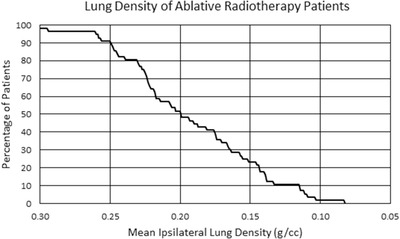
Reverse‐order cumulative histogram showing percentage of institutional ablative lung radiotherapy patients having a given mean lung density or lower

Lung density statistics for our institution's lung SABR patients is presented as a reverse‐order cumulative histogram in Figure 6, where the percentage of our patient population having a given mean lung density or lower is plotted as a function of mass density. Approximately 98% of our patients had a mean ipsilateral lung density of ≤0.30 g/cm^3^, 52% had a mean density ≤0.20 g/cm^3^, 23% had a mean density ≤0.15 g/cm^3^, and 2% had a mean density ≤0.10 g/cm^3^. The mean and minimum values for the sampled population were 0.19 and 0.07 g/cm^3^, respectively.

## 4 DISCUSSION

In this study, we have correlated combinations of various tissue characteristics with levels of tumor dose reduction, some of which may compromise the therapeutic intent of a course of ablative lung radiotherapy treatment when planned using the AAA. Specifically, we have investigated the interplay of lung tissue density, tumor density, tumor diameter, and beam energy on the ability of AAA to accurately predict the delivery of a therapeutically ablative dose. As part of this, we also validated the AcurosXB algorithm as an approximate ground‐truth representation of the underlying dose distribution even in extremely low‐density media.

The practical difference in tissue heterogeneity handling between AAA and AcurosXB becomes negligible for a mean lung density of 0.25–0.30 g/cm^3^, above which their dose calculation converges within a 2*σ* range for all studied variables. A lung density of 0.20 g/cm^3^ is a transitional point where deficits in tumor coverage begin to occur but are not likely to compromise treatment efficacy. No scenarios with a lung density of ≥0.20 g/cm^3^ resulted in a loss of 100‐Gy (BED10) coverage to the CTV or a CTV D95% result of less than the nominally prescribed dose, despite AAA overestimating the true delivered dose by as much as 16% in these scenarios. Validity of this result beyond our institution presumes the use of a sufficiently similar treatment planning technique that results in a coverage of the CTV at approximately the 115% dose level.

AAA and AcurosXB calculations diverge sharply as lung density decreases below 0.15 g/cm^3^, particularly for small tumors with a diameter of ≤10 mm. Within these tissue characteristic regions, there is an increasing risk of AAA grossly overestimating the tumor dose, with certain combinations of tissue variables resulting in a 20%–40% or a greater loss of 100‐Gy (BED10) dose coverage to the CTV depending on the chosen fractionation.

Tumor density was not found to be a large contributing factor to overall concerns with the use of AAA. Although correlated with tumor dose reduction the magnitude of the effect is comparatively small. A tumor density of 0.30 g/cm^3^ results in less than a 5% degradation of tumor coverage compared to densities in the more typical 0.65–1.00 g/cm^3^ range. Only in combination with a very low lung density and small tumor diameter does the lowest tumor density lead to a tumor coverage degradation of more than 5% relative to higher density tumors, and in such scenarios, this contribution remains negligible relative to the overall dose reduction.

Further, the use of a 10‐MV photon beam energy exacerbates the dose overestimation issue in these problematic tissue regions. For lung densities ≤0.15 g/cm^3^ in combination with tumor diameters ≤15 mm, the use of 10‐MV beam energy resulted in an average additional 21% loss of 100‐Gy (BED10) dose coverage to the CTV compared to the same plans generated with 6 MV.

There are compelling efficiency arguments for the use of a 10‐MV FFF beam when treating lung tumors to a high fraction dose, particularly if respiratory gating is being utilized. For the Varian Truebeam treatment platform, the choice of 10‐MV FFF with a 2400‐MU/min dose rate could result in a 40%–75% reduction in beam‐on time for a dose‐rate limited treatment compared to 6 MV (non‐FFF), 6‐MV FFF, or 10‐MV (non‐FFF) that have maximum dose rates in the range of 600–1400 MU/min. This time reduction could be highly beneficial from a patient compliance, satisfaction, and throughput perspective. However, if AAA is utilized for planning then the choice would have to be carefully weighed against the potential for unacceptable dosimetric deficiencies given each specific patient's tissue characteristics within the volume being irradiated.

The described trend of AAA overestimating tumor dose in these scenarios likely also has broader implications on the treatment plan's robustness against errors in patient setup, deficiencies in motion management, or inaccuracies in the reproduction of the plan by the treatment delivery system. If a gross overestimation of delivered dose by AAA brings the nominal treatment plan nearer to (but not exceeding) the point of therapeutic failure, then the permissible uncertainty in the delivery itself becomes correspondingly smaller. Although these treatment delivery uncertainties should be tightly controlled—particularly in ablative applications—they may play an unexpectedly greater role in the therapeutic outcome if the true baseline dose associated with the plan is sufficiently degraded from expectation.

The major limitation of this study was the use of discrete, uniform, and somewhat coarsely spaced simulated values for the various tissue variables. Actual lung and tumor volumes are heterogeneous and may have adjacent or embedded vasculature that impacts the local effective density within the irradiated region. Ideally this study could have been performed using only clinical cases that span the range of investigated tissue characteristics. However, this was not feasible in order to ensure sufficient sampling throughout the range of all studied variables. The presentation of data from clinical cases in Table 1 demonstrates that our clinical results largely fall within a reasonable range of this study's predicted values, but that ultimately the presented results must be taken as trends rather than exact results to be extrapolated to specific clinical cases.

Additionally, it should be noted that the treatment planning methodology of this study assumed the clinical treatment process of the author's institution where the GTV is immobilized via a reproducible visually coached breath‐hold rather than the generation of a motion‐encompassing internal target volume (ITV). Institutions using the ITV approach would likely have more lung tissue within the treatment field, which may change the magnitude of the effects demonstrated in this study for scenarios involving near‐minima lung density. Further, intrafraction tumor motion within the ITV would be an additional confounding factor for determination of the true tumor dose, but the underlying gross deficiencies of the AAA calculation in these specific tissue environments would continue to be present.

It is important to note that pretreatment quality assurance measurements in a commercial 2D or 3D array would not be expected to identify these deficiencies in the clinical treatment plan as these measurements are generally performed in devices of higher and nearly homogeneous density. An independent monitor unit verification could potentially identify these issues if the secondary algorithm had sufficiently different density handling than AAA. However, an institution having a secondary verification algorithm with more robust handling of heterogeneous tissue environments than their primary algorithm would be unusual, and it is not clear how such an institution could rectify the underlying issue if they did not have access to a more robust primary algorithm.

Additionally, standardized phantoms used for institutional credentialing for lung radiotherapy studies are fabricated with materials that have physical properties within the convergence region of the AAA and AcurosXB algorithms. The Imaging and Radiation Oncology Core anthropomorphic lung–thorax phantom uses a lung tissue surrogate with a density of approximately 0.30 g/cm^3^ and a tumor surrogate with density and axial‐plane diameter of approximately 1.00 g/cm^3^ and 3 cm, respectively. Under these conditions, an appropriately commissioned AAA beam model would be expected to perform to the same approximate standard as AcurosXB even if there could be major deficiencies in tissue heterogeneity handling for a subset of patients enrolled in those studies. Stratification of enrolled patients based on relevant tissue characteristics may be reasonable in order to establish a safe limit on the use of algorithms with these limitations.

## 5 CONCLUSIONS

The accuracy of the AAA rapidly diminishes within very low‐density lung tissue environments (≤0.15 g/cm^3^), particularly in combination with small tumors (≤15‐mm diameter) and when using a 10‐MV photon energy. Under these conditions, the delivered dose becomes severely overestimated by AAA and, in some scenarios, may result in a clinically unacceptable reduction in biologically effective dose delivered to the CTV, particularly with less aggressive hypofractionated regimens where the true 100‐Gy (BED10) tumor volume coverage may be a small fraction of the treatment planning system prediction. Although the fraction of impacted patients is likely small, it is nonzero and therefore crucial to consider. A careful review of these tissue characteristics on the CT dataset used for planning is critical if using AAA, and options for mitigation (such as the use of lower photon energy) should be considered as needed.

## CONFLICT OF INTEREST

None.

## AUTHOR CONTRIBUTION

All listed authors contributed substantially to the study design and execution, and the drafting and reviewing of the manuscript. Each author approved the final submitted version of the manuscript.

## References

[1] Abel S , Hasan S , Horne ZD , Colonias A , Wegner RE . Stereotactic body radiation therapy in early‐stage NSCLC: historical review, contemporary evidence and future implications. Lung Cancer Manag. 2019;8(1):LMT09. 10.2217/lmt-2018-0013. PMID: 31044018; PMCID: PMC6488937. 31044018 PMC6488937

[2] Eclipse Photon and Electron Algorithms Reference Guide (Document ID P1020505002B). Varian Medical Systems, Inc.; 2017.

[3] Das IJ , Ding GX , Ahnesjö A . Small fields: nonequilibrium radiation dosimetry. Med Phys. 2008;35(1):206‐215. 10.1118/1.2815356. PMID. 18293576. 18293576

[4] Imaging and Radiation Oncology Core . TPS (Treatment Planning Systems) approved for calculation of dose with heterogeneities. Retrieved September 30th, 2021. http://irochouston.mdanderson.org/RPC/Services/Anthropomorphic_%20Phantoms/TPS%20‐%20algorithm%20list%20updated.pdf

[5] Glenn MC , Peterson CB , Followill DS , Howell RM , Pollard‐Larkin JM , Kry SF . Reference dataset of users' photon beam modeling parameters for the Eclipse, Pinnacle, and RayStation treatment planning systems. Med Phys. 2020;47(1):282‐288. 10.1002/mp.13892. Epub 2019 Nov 15. PMID: 31667870; PMCID: PMC6980266. 31667870 PMC6980266

[6] Kroon PS , Hol S , Essers M . Dosimetric accuracy and clinical quality of Acuros XB and AAA dose calculation algorithm for stereotactic and conventional lung volumetric modulated arc therapy plans. Radiat Oncol. 2013;8:149. 10.1186/1748-717X-8-149 23800024 PMC3723919

[7] Mohatt DJ , Ma T , Wiant DB , et al. Technical and dosimetric implications of respiratory induced density variations in a heterogeneous lung phantom. Radiat Oncol. 2018;13:165. 10.1186/s13014-018-1110-2 30180894 PMC6124019

[8] Hedin E , Chakarova R , Bäck A . Implementation of Acuros XB in treatment planning of SBRT of lung cancer. Ann Radiat Ther Oncol. 2017;1(1):1009.

[9] Yan C , Combine AG , Bednarz G , et al. Clinical implementation and evaluation of the Acuros dose calculation algorithm. J Appl Clin Med Phys. 2017;18(5):195‐209. 10.1002/acm2.12149. Epub 2017 Aug 20. PMID: 28834214; PMCID: PMC5875823. 28834214 PMC5875823

[10] Rana S , Rogers K , Pokharel S , Cheng C . Evaluation of Acuros XB algorithm based on RTOG 0813 dosimetric criteria for SBRT lung treatment with RapidArc. J Appl Clin Med Phys. 2014;15(1):4474. 10.1120/jacmp.v15i1.4474. PMID: 24423844; PMCID: PMC5711238. 24423844 PMC5711238

[11] Hoffmann L , Jørgensen MB , Muren LP , Petersen JB . Clinical validation of the Acuros XB photon dose calculation algorithm, a grid‐based Boltzmann equation solver. Acta Oncol. 2012;51(3):376‐385. 10.3109/0284186X.2011.629209. Epub 2011 Nov 7. PMID: 22060134. 22060134

[12] Shiraishi S , Fong de Los Santos LE , Antolak JA , et al. Phantom verification of AAA and Acuros dose calculations for lung cancer: do tumor size and regression matter?. Pract Radiat Oncol. 2019;9(1):29‐37. 10.1016/j.prro.2018.06.008. Epub 2018 Aug 20. PMID: 30138746. 30138746

[13] Sullivan R , Popple R . The clinical implementation and dosimetric comparison of Acuros‐XB (v15) and AAA (v13) for stereotactic body radiotherapy for lung cancer. Poster Presented at the 2020 Joint AAPM COMP Virtual Meeting.

[14] Ojala JJ , Kapanen MK , Hyödynmaa SJ , Wigren TK , Pitkänen MA . Performance of dose calculation algorithms from three generations in lung SBRT: comparison with full Monte Carlo‐based dose distributions. J Appl Clin Med Phys. 2014;15(2):4662. 10.1120/jacmp.v15i2.4662. PMID: 24710454; PMCID: PMC5875463. 24710454 PMC5875463

[15] Chetty IJ , Devpura S , Liu D , et al. Correlation of dose computed using different algorithms with local control following stereotactic ablative radiotherapy (SABR)‐based treatment of non‐small‐cell lung cancer. Radiother Oncol. 2013;109(3):498‐504. 10.1016/j.radonc.2013.10.012. Epub 2013 Nov 11. PMID: 24231237. 24231237

[16] Liu H‐W , Nugent Z , Clayton R , Dunscombe P , Lau H , Khan R . Clinical impact of using the deterministic patient dose calculation algorithm Acuros XB for lung stereotactic body radiation therapy. Acta Oncol. 2014;53(3):324‐329. 10.3109/0284186X.2013.822552 23957683

[17] Biernacki W , Redpath AT , Best JJ , MacNee W . Measurement of CT lung density in patients with chronic asthma. Eur Respir J. 1997;10(11):2455‐2459. 10.1183/09031936.97.10112455. PMID. 9426078. 9426078

[18] Niroomand‐Rad A , Chiu‐Tsao ST , Grams MP , et al. Report of AAPM Task Group 235 radiochromic film dosimetry: an update to TG‐55. Med Phys. 2020;47(12):5986‐6025. 10.1002/mp.14497. Epub 2020 Oct 30. PMID: 32990328. 32990328

[19] Corso CD , Park HS , Moreno AC , et al. Stage I lung SBRT clinical practice patterns. Am J Clin Oncol. 2017;40(4):358‐361. 10.1097/COC.0000000000000162. PMID. 25503436. 25503436

[20] Hiraoka M , Matsuo Y , Nagata Y . Stereotactic body radiation therapy (SBRT) for early‐stage lung cancer. Cancer Radiother. 2007;11(1–2):32‐35. 10.1016/j.canrad.2006.11.001. Epub 2006 Dec 8. PMID: 17158081. 17158081

[21] Onishi H , Shirato H , Nagata Y , et al. Stereotactic body radiotherapy (SBRT) for operable stage I non‐small‐cell lung cancer: can SBRT be comparable to surgery?. Int J Radiat Oncol Biol Phys. 2011;81(5):1352‐1358. 10.1016/j.ijrobp.2009.07.1751. Epub 2010 Jul 16. PMID: 20638194. 20638194

[22] Nuyttens JJ , van der Voort van Zyp NC , Praag J , et al. Outcome of four‐dimensional stereotactic radiotherapy for centrally located lung tumors. Radiother Oncol. 2012;102(3):383‐387. 10.1016/j.radonc.2011.12.023. Epub 2012 Jan 20. PMID: 22265734. 22265734

[23] Abel S , Hasan S , Horne ZD , Colonias A , Wegner RE . Stereotactic body radiation therapy in early‐stage NSCLC: historical review, contemporary evidence and future implications. Lung Cancer Manag. 2019;8(1):LMT09. 10.2217/lmt-2018-0013. Published 2019 Feb 27. 31044018 PMC6488937

[24] Videtic GM , Chang JY , Chetty IJ , et al. ACR appropriateness Criteria® early‐stage non‐small‐cell lung cancer. Am J Clin Oncol. 2014;37(2):201‐207. 10.1097/COC.0000000000000013 25180631

[25] Prezzano KM , Ma SJ , Hermann GM , Rivers CI , Gomez‐Suescun JA , Singh AK . Stereotactic body radiation therapy for non‐small cell lung cancer: a review. World J Clin Oncol. 2019;10(1):14‐27. 10.5306/wjco.v10.i1.14 30627522 PMC6318482

